# Dose Adjustment Model of Paliperidone in Patients With Acute Schizophrenia: A *post hoc* Analysis of an Open-Label, Single-Arm Multicenter Study

**DOI:** 10.3389/fpsyt.2021.723245

**Published:** 2021-08-23

**Authors:** Tianmei Si, Ling Sun, Yilong Zhang, Lili Zhang

**Affiliations:** ^1^Peking University Sixth Hospital (Institute of Mental Health), Beijing, China; ^2^NHC Key Laboratory of Mental Health (Peking University) & National Clinical Research Center for Mental Disorders (Peking University Sixth Hospital), Beijing, China; ^3^Tianjin Anding Hospital, Tianjin, China; ^4^Xian Janssen Pharmaceuticals, Beijing, China

**Keywords:** acute, schizophrenia, paliperidone, dose adjustment, decision tree

## Abstract

This study aimed to investigate the factors that influenced the clinicians to adjust the paliperidone dose in the acute phase of schizophrenia. This was a *post hoc* study of an 8-week, open-label, single-arm multicenter trial which evaluated the efficacy, safety, and tolerability of flexible doses of paliperidone ER (3–12 mg/day) in patients with acutely exacerbated schizophrenia. Patients were divided into groups according to the dose at week 8 (3, 6, and 9–12 mg). The responder was defined as the reduction percentage in the Positive and Negative Syndrome Scale (PANSS) total score of ≥30%. According to the chi-squared automatic interaction detection algorithm, decision tree models predicting an increase in the dose of paliperidone ER were established. A decision tree, based on 4-week Marder positive factor, Clinical Global Impression (CGI), and BMI, was established to guide the dose adjustments of paliperidone ER in the acute phase of schizophrenia. The multivariable logistic regression analysis showed that lower age at onset, higher baseline PANSS positive subscale score, and lower baseline Personal and Social Performance Scale (PSP) score were significant predictors of increased dose in responders. Patients with young-onset age, severe baseline symptoms, and poor function are more likely to benefit from high dosage.

## Introduction

Schizophrenia is a chronic and disabling mental disorder with serious physical, social, and economic consequences (1). Many antipsychotic agents have been developed, showing good effects on positive symptoms (2–4). Paliperidone is an antipsychotic drug for schizophrenia. It is an active metabolite of risperidone (9-OH risperidone) and has almost the same pharmacological profile, with a high affinity for the dopamine D2 receptor and the serotonin 5-hydroxytryptamine 2A receptor (5). Early studies suggested paliperidone extended-release (ER) with a starting dose of 6 mg once daily without titration (1, 6). However, it is currently admitted that titration is necessary for some patients (7). Still, adjusting the dose of paliperidone ER in patients with acute schizophrenia is poorly understood.

Decision-making is one of the most important roles of clinicians (8, 9). Clinically, a weighted thought process leads to treatment decisions (8, 9). Far too often, decisions are made with limited knowledge, leading to significantly different approaches being advocated for the same clinical scenario (8, 9). Therefore, decision trees can be useful for management standardization in conditions that range from routine to highly complex (10, 11). Building a decision tree involves identifying all the available choices and the respective potential outcomes (12, 13).

Although increasing the dose of antipsychotics may be somewhat questionable in general, many clinicians often choose this option in case of a suboptimal response (14). There are few reports on decision tree analyses that detail the predictors of dose adjustment for paliperidone. The use of a decision tree for dose adjustment could facilitate and standardize paliperidone dose adjustment. An 8-week, open-label, single-arm multicenter study supported efficacy, safety, and tolerability profiles of flexible doses (3–12 mg/day) of paliperidone ER in Chinese patients with acutely exacerbated schizophrenia (15). A *post hoc* analysis of that study was conducted to investigate factors influencing the clinician's decision to adjust paliperidone dose in the acute phase of schizophrenia. In addition, baseline variables influencing the optimal dose for patients in the acute phase of schizophrenia were evaluated.

## Methods

### Data Source

The original study was an 8-week, open-label, single-arm multicenter trial that evaluated the efficacy, safety, and tolerability of flexible doses of paliperidone ER (3–12 mg/day) in patients with acutely exacerbated schizophrenia (15). The original study was conducted at 20 sites in China. The study protocol and amendments were reviewed by the Shanghai Mental Health Center Institutional Review Board. Written informed consent was obtained from each participant.

Patients aged ≥18 years with a diagnosis of acute schizophrenia (Diagnostic and Statistical Manual of Mental Disorders, 4th Edition criteria) and a Positive and Negative Syndrome Scale (PANSS) total score ≥70 at baseline were enrolled in the original study. The participants volunteered to stay in the hospital and undergo treatment for acute schizophrenic symptoms for the first 7 days. The included women were menopausal for at least 1 year before enrollment; otherwise, they had undergone surgical sterilization, were abstinent, or performed effective contraception (oral contraceptive, injectable contraceptives, intrauterine device, double-barrier methods of contraception, birth-control patch, or spouse sterilization). In women with child-bearing potential, a urine pregnancy test had to be negative during screening. Patients with a diagnosis of substance abuse (current or within the previous 6 months), a history of tardive dyskinesia or neuroleptic malignant syndrome, apparent suicide tendency, or violent behavior were excluded. The present *post hoc* analysis included participants who completed the 8-week trial.

### Treatment

The participants were hospitalized within the first 7 days after study initiation. Follow-up visits were scheduled in weeks 2, 4, and 8. Changes in PANSS total score, PANSS Marder factors, Personal and Social Performance Scale (PSP), Clinical Global Impression (CGI), and treatment satisfaction were assessed at every visit. Treatment satisfaction was assessed by a numeric scale of 1–5 (1 = “extremely satisfied;” 2 = “satisfied;” 3 = “neither satisfied nor dissatisfied;” 4 = “dissatisfied;” 5 = “not at all satisfied”). All investigators were trained in the use of the scales used in the study. All participants received 6 mg/day of paliperidone ER at the beginning, and the dose could be adjusted at weeks 2 and 4 in the follow-up visit, based on the participant's response. The last dose adjustment was performed on week 4. The dose of paliperidone ER varied from 3 to 12 mg/day throughout the trial. The flexible dose allowed the investigators to adjust doses for each participant based on clinical indications and drug tolerability. Considering the safety of participants, the doses were decreased on-demand.

### Grouping

Participants were grouped according to the dose at week 8 (3, 6, and 9–12 mg/day). The reduction percentage of the PANSS total score was dichotomized as ≥30% and <30%. In order to investigate the effect of the early dose pattern on the effectiveness of paliperidone ER, participants were divided into four subgroups according to the prescribed dose and the reduction in PANSS total score: (1) “unadjusted dose responder subgroup,” including participants with a PANSS total score reduction ≥30% and a dose of 6 mg/day at week 8; (2) “unadjusted dose non-responder subgroup,” including participants with a PANSS total score reduction <30% and a dose of 6 mg/day at week 8; (3) “increased dose responder subgroup,” including participants with a PANSS total score reduction ≥30% and a dose >6 mg/day at week 8; (4) “increased dose non-responder subgroup,” including participants with a PANSS total score reduction <30% and a dose >6 mg/day at week 8.

### Statistical Analysis

Decision tree analysis was performed with SPSS Decision Trees Version (IBM, Armonk, NY, USA). According to the chi-squared automatic interaction detection (CHAID) algorithm, decision tree models predicting an increase in paliperidone ER dose were established. CHAID is a non-parametric procedure that makes no assumptions of the underlying data and does not require any assumptions about the statistical distribution of the examined predictor variables (16). The CHAID algorithm determines how continuous and/or categorical independent variables can be combined to predict a binary outcome based on the “if-then” logic and by portioning each independent variable into mutually exclusive subsets based on the homogeneity of the data. We did not limit the number of branches (depth) of the decision tree. Because the visit on week 4 was the last dose adjustment opportunity during the study period, the decision tree was trained using baseline and week 4 visit variables. The decision tree selected variables that discriminated the groups. The decision-making process for dose adjustment was simply the rule extracted from the decision tree that achieved the highest discrimination.

Depending on whether changes between baseline and endpoint met a Gaussian distribution, Student's *t*-test or the Wilcoxon signed-rank test was performed for baseline continuous data. Baseline categorical data were analyzed by the chi-square test or Fisher's exact test. Baseline covariates with *P* < 0.05 in univariable analysis were included in the multivariable stepwise logistic regression model. All statistical analyses were performed with SPSS 17.0 (SPSS Inc., Chicago, IL). Receiver operating characteristics (ROC) curves were used to determine the best cut-off points of the identified factors. Two-sided *P* < 0.05 was considered statistically significant.

## Results

### Participant Characteristics

Of the 608 participants enrolled in the original study, 500 (82.2%) completed the 8-week trial and were included in the present *post hoc* analysis. The mean participant age at baseline was 32.2 ± 11.5 years; 50.0% (250/500) of them were female, and 50.0% (250/500) were male. All participants received 6 mg/day of paliperidone ER at the beginning of the study. The mean dose was 7.8 ± 2.2 mg/day during the 8-week study, with a daily dose range of 3–12 mg. After initiation, paliperidone ER's daily dose was increased in 54.6% of participants and decreased in 4.2%. Baseline PANSS total score, PSP score are shown in Table 1. The combined use of anti-EPS drugs during the study in each group were shown in Table 2. Because the sample size of the 3-mg group was too small (*n* = 21), it was not included in subsequent statistical analyses.

**Table 1:** Baseline characteristics of the participants.

**Variable**	**3 mg**	**6 mg**	**9–12 mg**	**All**
	**(*n* = 21)**	**(*n* = 206)**	**(*n* = 273)**	**(*n* = 500)**
Sex				
Male	8 (38.1)	105 (51.0)	137 (50.2)	250 (50.0)
Female	13 (61.9)	101 (49.0)	136 (49.8)	250 (50.0)
Age (years)	28.8 ± 11.5	33.0 ± 11.3	31.9 ± 11.6	32.2 ± 11.5
Duration of disease (years)	3.7 ± 4.7	7.8 ± 9.2	7.7 ± 8.6	7.6 ± 8.8
PANSS total score	86.1 ± 13.4	87.4 ± 12.2	91.4 ± 14.3	89.5 ± 13.6
PSP score	52.6 ± 12.6	47.0 ± 12.4	41.1 ± 12.4	44.0 ±1 2.8

*Data are mean ± standard deviation or n (%).; PANSS, Positive and Negative Syndrome Scale; PSP, Personal and Social Performance Scale*.

**Table 2:** Anti-EPS drug* combination during the study in each group.

**Combined Anti-EPS drug**	**3 mg**	**6 mg**	**9–12 mg**	***P*-value (6 mg vs. 9–12 mg)**
Baseline, *n* (%)				0.5717
*n* (N missing)	21 (0)	206 (0)	273 (0)	
No	21 (100.00)	175 (84.95)	236 (86.45)	
Yes	0	31 (15.05)	37 (13.55)	
Day 4, *n* (%)				0.1071
*n* (N missing)	21 (0)	206 (0)	273 (0)	
No	19 (90.48)	164 (79.61)	200 (73.26)	
Yes	2 (9.52)	42 (20.39)	73 (26.74)	
Week1, *n* (%)				0.2468
*n* (N missing)	21 (0)	206 (0)	273 (0)	
No	17 (80.95)	159 (77.18)	198 (72.53)	
Yes	4 (19.05)	47 (22.82)	75 (27.47)	
Week 2, *n* (%)				0.2846
*n* (N missing)	21 (0)	206 (0)	273 (0)	
No	17 (80.95)	159 (77.18)	199 (72.89)	
Yes	4 (19.05)	47 (22.82)	74 (27.11)	
Week 4, *n* (%)				0.4889
*n* (N missing)	21 (0)	206 (0)	273 (0)	
No	17 (80.95)	142 (68.93)	180 (65.93)	
Yes	4 (19.05)	64 (31.07)	93 (34.07)	
Combined Anti-EPS drug at week 8, *n* (%)				0.4832
*n* (Nmissing)	21 (0)	206 (0)	273 (0)	
No	17 (80.95)	156 (75.73)	199 (72.89)	
Yes	4 (19.05)	50 (24.27)	74 (27.11)	

*EPS, extrapyramidal symptoms*.

### Decision Tree

**Figure 1:**
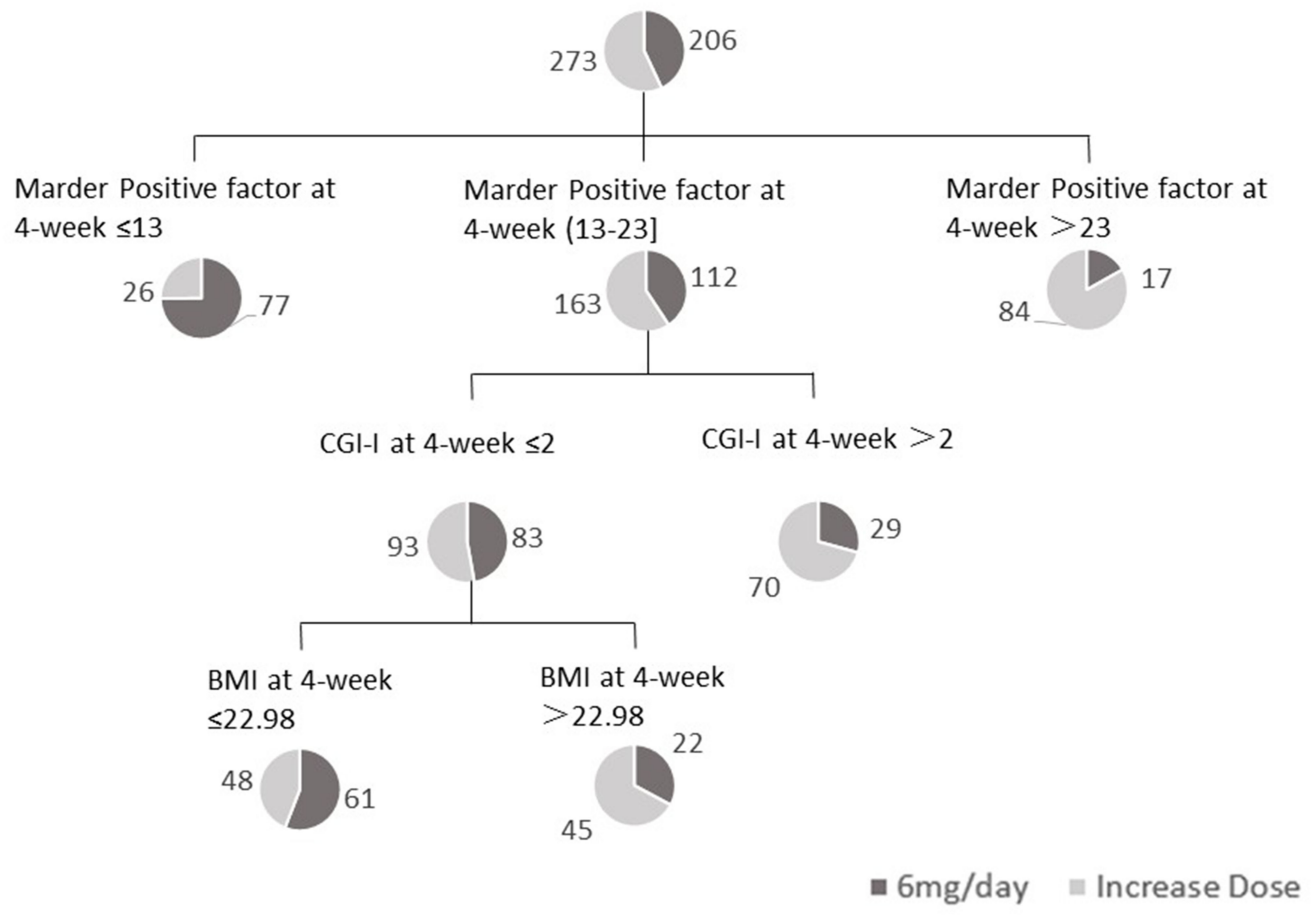
A decision tree for the prediction of paliperidone ER dose increase. CGI-I, Clinical Global Impression-Improvement; BMI, body mass index.

As shown in Figure 1, a decision tree was established using baseline and 4-week data for various factors [sex, age, duration of disease, age at onset, PANSS total score, PANSS Marder factors, PSP score, CGI-Severity [CGI-S], treatment satisfaction and BMI]. The order of splitting variables was determined according to the strength of their relationships to dose change, based on the CHAID algorithm. As shown in Figure 1, Marder positive factor score at week 4 was selected as the first splitting variable. At a Marder positive factor score >23, the dose was increased in 83.2% (84/101) of all participants. At a Marder positive factor score <13, the dose was maintained at 6 mg in 74.8% (77/103) of participants. In participants with a Marder positive factor score of 13–23 at week 4, CGI-Improvement (CGI-I) was selected as the second splitting variable. Participants with minimal improvement, no change, or worsening according to CGI-I (>2) were more likely (70.7%; 70/99) to be treated with a higher paliperidone ER dose. In participants with CGI-I ≤2 at week 4, BMI was selected as the third splitting variable. With a BMI at week 4 >23.0 kg/m^2^ [based on the cut-off in Asians according to the World Health Organization (17)], the dose was increased in 67.2% (45/67) of participants.

### Multivariable Logistic Regression Analysis

Significant baseline predictors identified using a stepwise logistic regression models are shown in Table 3. The two subgroups of responders (6 vs. 9–12 mg/day) were primarily analyzed. In univariable analysis, baseline PANSS total score, baseline PANSS positive subscale score, baseline PANSS anxiety/depression subscale score, age at onset, baseline CGI-S, and baseline PSP score showed significant differences between the two subgroups. These significant different variables were taken into Multivariable analysis. Multivariable analysis showed that significant independent predictors of dose increase in responders were lower age at onset [odds ratio [OR] = 0.966, 95% confidence interval [CI]: 0.938–0.994, *P* = 0.018], higher baseline PANSS positive subscale score (OR = 1.048, 95%CI: 1.003–1.096, *P* = 0.037), and lower baseline PSP score (OR = 0.972, 95%CI: 0.953–0.993, *P* = 0.007).

**Table 3:** Baseline predictors identified using exploratory analyses with explanatory variables entered into the multivariable stepwise logistic regression model.

**Model**	**Significant predictor**	***P***	**OR**	**95%CI**
Responders (6 mg/day vs. 9–12 mg/day)	Age at onset	0.018	0.966	0.938–0.994
	Baseline PANSS positive subscale score	0.037	1.048	1.003–1.096
	Baseline PSP score	0.007	0.972	0.953–0.993
Responders vs. non-responders (both with 6 mg/day)	Duration of disease	0.011	0.957	0.926–0.990
Responders vs. non-responders (both with 9–12 mg/day)	Duration of disease	0.043	0.970	0.941–0.999
	Baseline PANSS total score	0.031	1.023	1.002–1.043

*PANSS, Positive and Negative Syndrome Scale; PSP, Personal and Social Performance Scale*.

ROC curve analysis showed cut-off values of 24.5 years for age at onset, 28.5 for baseline PANSS positive subscale score, and 42.5 for baseline PSP score. The same statistical method was used between responders and non-responders at the same dose level. In the 6 mg/day group, a longer duration of disease (OR = 0.957, 95%CI: 0.926–0.990, *P* = 0.011) was an independent predictor of no-response, with a cut-off value of 4.5 years. In the 9–12 mg/day group, a longer duration of disease (OR = 0.970, 95%CI: 0.941–0.999, *P* = 0.043) and higher baseline PANSS total score (OR = 1.023, 95%CI: 1.002–1.043, *P* = 0.031) were independent predictors of no-response, with cut-off values of 7.5 years and 87.5, respectively.

## Discussion

The results showed that 41.2% of participants who completed the 8-week trial remained on the initial dose of 6 mg/day, while the dose was increased in 54.6% of participants. Although the recommended initiation dose of paliperidone ER is 6 mg/day (1, 6), studies suggested that a dose higher than 6 mg could be more effective in selected patients (7, 18–20). According to a study in which schizophrenia cases were stabilized during an 8-week run-in phase and then observed for symptom recurrence after the stabilization phase, 45% and 47% of all patients received paliperidone ER at 9 and 12–15 mg/day, respectively, during the flexible dosing (3-15 mg/day) run-in phase (18). According to another study of pooled data from three 52-week, open-label studies in which all patients received a flexible-dose of paliperidone ER (3–15 mg/day), with a starting dose of 9 mg/day, the mean and mean modal daily doses of paliperidone ER were 10.0 ± 2.4 and 10.7 ± 2.9 mg, respectively (19). In these clinical trials, there was a tendency for investigators to titrate upward from the starting dose of 9 mg/d. Another pooled data analysis from three 6-week, placebo-controlled studies suggested that the fixed paliperidone ER dose of 9 mg/day had a greater completion rate (66 vs. 56%) and a lower or similar dropout rate due to adverse events (4 vs. 7%) compared with 6 mg/day, despite higher EPS-related adverse events (25 vs. 10%) (21). These results suggest that a paliperidone ER dose higher than 6 mg might be needed for some patients with schizophrenia.

Decision trees constitute useful tools for clinical decision-making and are appealing to clinicians because they represent data and allow treatment standardization (21–24). Using data mining, a patient's data can be extracted and analyzed together with the clinician's decisions (25). In the present study, the decision tree model was used to simulate the thinking factors used by clinicians during decision-making. The root node was split by the Marder positive factor, CGI-I, and BMI at 4-week. In other words, physicians tended to increase the dose of paliperidone in patients with severe positive symptoms, less clinical mental progress, and higher BMI. The splitting variables of severe positive symptoms or less clinical mental progress are quite aligned with clinical guideline recommendations and clinical practice, which means increase dosage when patients did not get symptom control or significant improvement. Precious publication also reported BMI as a predictor factor for higher paliperidone dose in acute schizophrenia patients (22). According to the decision tree, physicians paid more attention to the state of the patient when deciding whether to increase the dose of paliperidone in clinical practice.

The participants were divided into four subgroups according to dose increase and changes in PANSS total score, and baseline predictors of paliperidone ER dose and treatment response were identified by logistic regression analysis. According to an analysis of the “unadjusted dose responder” and “increased dose responder” groups, participants with younger age at the first onset, higher baseline PANSS positive subscale score, and lower baseline PSP score would suppose to have better therapeutic effect using higher doses. Using stepwise logistic regression analysis, Heres et al. (22) found that higher BMI was a significant predictor of a dose >6 mg/day. The significant predictors of a dose of 9 mg/day were elevated BMI and a higher number of hospitalizations during the previous 12 months (22). However, the latter study did not analyze the different dose groups for treatment response, and the obtained predictors could not predict better outcomes through different doses. Age at onset does not change with treatment and might be closely related to treatment outcome and relapse in schizophrenic patients. A systematic review and meta-analysis suggested significant associations of younger age at onset with higher number of hospitalizations (number of studies, *n* = 9; correlation, *r* = 0.17), increased amounts of negative symptoms (*n* = 7; *r* = 0.14), elevated number of relapses (*n* = 3; *r* = 0.11), poorer social/occupational functioning (*n* = 12; *r* = 0.15), and poorer global outcome (*n* = 13; *r* = 0.14) (23). Overall, younger age at onset might result in poorer baseline functioning, especially regarding work and social adjustment, although such association may be diluted over time due to intervention programs. Therefore, for younger patients at onset, the dose should be increased to achieve more benefits. As for baseline PSP and PANSS positive subscale scores, since this study enrolled schizophrenia patients in the acute phase, dose increase should be considered for individuals with serious impairment of social function or severe positive symptoms in acute exacerbation. An analysis of the “unadjusted dose responder,” “unadjusted dose non-responder,” “increased dose responder,” and “increased dose non-responder” groups showed that a longer duration of disease was a significant predictor of worse treatment outcome. Thus, more attention should be paid to preventing relapse in clinical practice as patients with a longer duration of disease might not significantly benefit from dose increase.

The blood levels of the drug were not monitored in this study. This study mainly investigated whether relatively high doses (≥9 mg) should be provided and to which patients; thus, 4-week data were used as a reference, even though treatment response could be predicted as early as 2-weeks (24). In this study, symptom management was used as evidence for adjusting drug doses. In the actual clinical practice, besides symptom management, a drug blood level test could be very helpful to the dose decision procedure; currently, monitoring the blood levels of drugs is mainly used in patients with refractory diseases or problems after drug dose adjustment.

In this study, anti-EPS drugs were allowed; there were no significant differences between the 6 and 9–12 mg/day groups in the anti-EPS drug combination percentage during the study (Table 2). Still, the symptoms were managed after combined drug therapy, and no patient discontinued the treatment due to EPS in this study. Nevertheless, raising the dose in a patient with EPS, even if the EPS are being treated with an anti-EPS agent, may produce a worse outcome; two small sample size studies of risperidone showed that in patients with EPS, reducing dosages for relieving side effects may yield efficacy, which needs further investigation for a substantial conclusion (25, 26). Additionally, in both studies, patients could receive anti-EPS treatment.

In individuals with acute-phase schizophrenia, rapid control of symptoms is closely associated with patient outcomes (2–4). The decision tree established in this study could provide reference information and evidence for identifying patients that may require higher doses to control and stabilize their symptoms, therefore achieving an earlier control of the disease.

This study had some additional limitations. First, data interpretation was limited by the study design as an open-label, single-arm treatment trial, in addition to limitations inherent to secondary analyses of clinical trial data (27, 28). Paliperidone ER was up- and down-titrated (3–12 mg) once every 2-weeks during the study, based on the clinician's decision. The role of dose and titration in the early (at 2-weeks or earlier) response needs further study. Secondly, the short study period in the present analysis (first 4-weeks) hindered the assessment of the predictive values of these variables in long-term outcomes. Thirdly, in order to have a reasonable observe window to dose adjustment, we only analyzed subjects who completed the 8-week treatment period. Therefore, the effect of dropout was not considered. Fourthly, although predictors in the multivariable logistic analysis were statistically significant, 95%CIs were still close to 1.00. Therefore, these factors can only be used as a directional suggestion for evaluation and screening. Fifthly, the data were insufficient, and a reliable receiver operating characteristic analysis was not possible. Further studies assessing effectiveness and adherence are needed. Sixthly, some cut-off points for the decision tree were based on convenience. Finally, and most importantly, this study was heavily dependent on the characteristics of the study sample. Therefore, the generalizability is limited, and the obtained findings are applicable only to acutely exacerbated cases (who are likely to show notable positive symptoms) but not to patients with predominantly negative or cognitive symptoms.

In conclusion, a decision tree based on 4-week Marder positive factor, CGI-I, and BMI was developed to reflect the dose adjustment of paliperidone ER in the acute phase of schizophrenia. Patients with young age at onset, severe baseline symptoms, and poor function are more likely to benefit from high doses.

## Data Availability Statement

The original contributions presented in the study are included in the article/supplementary material, further inquiries can be directed to the corresponding author/s.

## Ethics Statement

The studies involving human participants were reviewed and approved by the Shanghai Mental Health Center Institutional Review Board. The patients/participants provided their written informed consent to participate in this study.

## Author Contributions

TS conceived and coordinated the *post hoc* study, designed, performed, analyzed the experiments, and wrote the paper. LS, YZ, and LZ carried out the data collection, data analysis, and revised the paper. All authors reviewed the results and approved the final version of the manuscript.

## Conflict of Interest

YZ and LZ were employees of Xian Janssen Pharmaceuticals at the time during which the work was undertaken. This work is a *post-hoc* analysis of a Xian Janssen sponsored phase 3 study R0776477-SCH-3034. TS has been a consultant and advisor to or has received honoraria and research grant/support from Xi'an-Janssen, Pfizer, Lundbeck and Otsuka. The remaining author declares that the research was conducted in the absence of any commercial or financial relationships that could be construed as a potential conflict of interest.

## Publisher's Note

All claims expressed in this article are solely those of the authors and do not necessarily represent those of their affiliated organizations, or those of the publisher, the editors and the reviewers. Any product that may be evaluated in this article, or claim that may be made by its manufacturer, is not guaranteed or endorsed by the publisher.
